# Supplementary material for: ASPP2 maintains the integrity of mechanically stressed pseudostratified epithelia during morphogenesis
Source: Nat Commun. 2022 Feb 17;13:941. doi: 10.1038/s41467-022-28590-4 (PMC8854694; doi:10.1038/s41467-022-28590-4)
Supplement: Supplementary file 1 — Supplementary Information [file 41467_2022_28590_MOESM1_ESM.pdf]

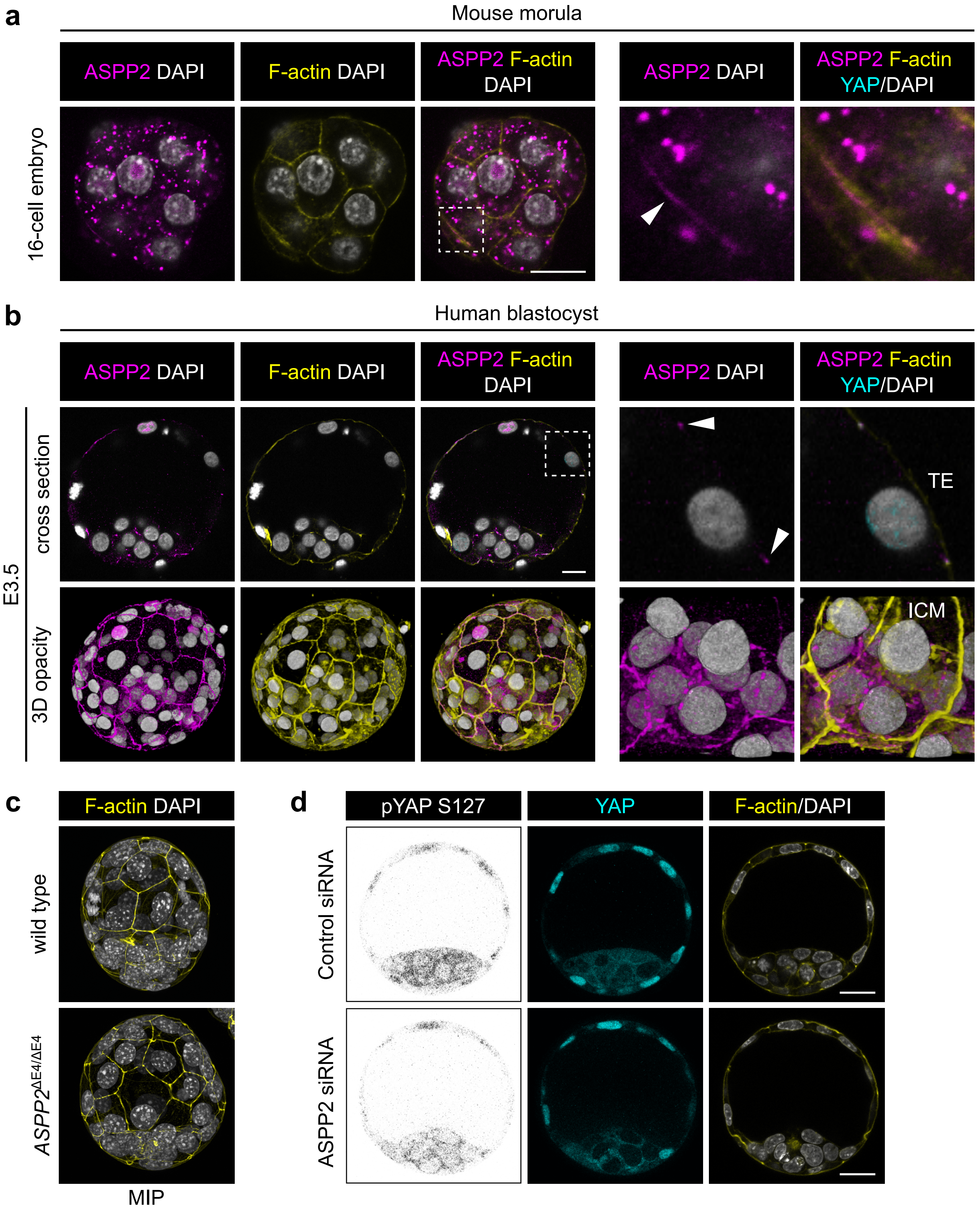

Supplementary Fig. 1 | ASPP2 is not required during preimplantation development. a, Localisation pattern of ASPP2 in 16-cell embryos (representative images from 4 embryos). A cross section through the equatorial plane of a representative embryo is shown. The F-actin cytoskeleton and nuclei were visualised using Phalloidin and DAPI, respectively. The dashed area is magnified on the right. The white arrowhead shows ASPP2 and F-actin colocalising at an apical junction between two outside cells. Scale bars: 20 µm (left panel) and 5 µm (right panel). **b**, Localisation pattern of ASPP2 in human blastocysts (representative images from 6 out of 10 embryos). The top panel shows a cross section through the equatorial plane of a representative embryo. The dashed area is magnified on the right to highlight the colocalisation between ASPP2 and F-actin at the level of apical junctions in the trophectoderm (white arrowheads). The bottom row shows a 3D opacity rendering of the same embryo in its totality (left panel) and a focus on its inner cell mass (right panel). TE: trophectoderm; ICM: Inner cell mass. Scale bar: 20 µm. **c**, F-actin is normally distributed at the apical junctions in the trophectoderm of *ASPP2*$^{\Delta E4/\Delta E4}$ embryos. Maximum intensity projections of representative wild type (representative image from 15 embryos) and *ASPP2*$^{\Delta E4/\Delta E4}$ (representative image from 3 embryos) embryos stained with Phalloidin and DAPI. **d**, ASPP2 knockdown in E3.5 embryos using siRNA targeting ASPP2 mRNA. Note that the localisation pattern of pYAP S127 was similar in control and ASPP2-depleted embryos. Representative images from 3 control siRNA-injected embryos and 4 ASPP2 siRNA-injected embryos. Scale bar: 20 µm.

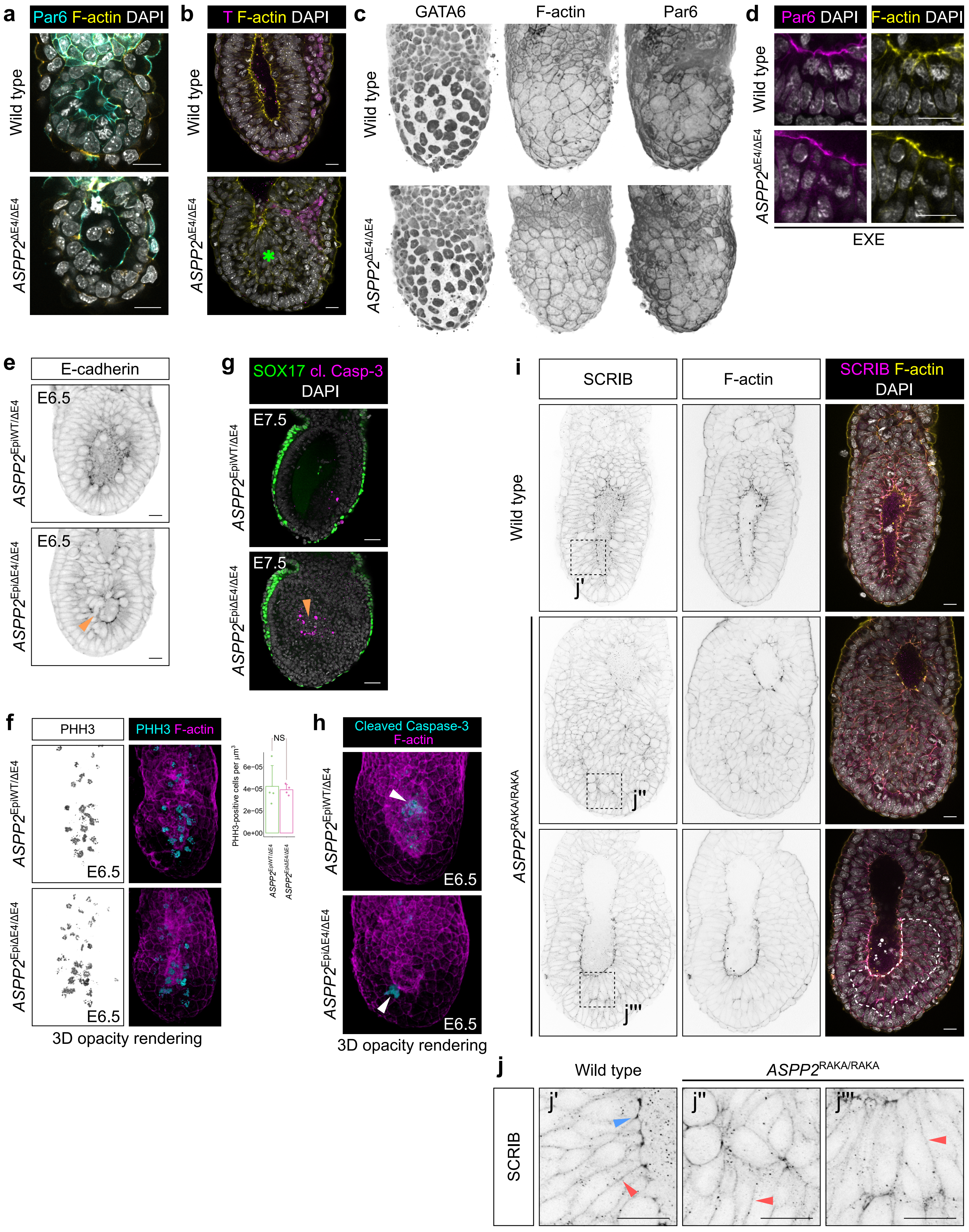
a
Par6 F-actin DAPI
Wild type
ASPP2ΔE4/ΔE4
b
T F-actin DAPI
Wild type
ASPP2ΔE4/ΔE4
c
GATA6
F-actin
Par6
Wild type
ASPP2ΔE4/ΔE4
d
Par6 DAPI
F-actin DAPI
Wild type
ASPP2ΔE4/ΔE4
EXE
e
E-cadherin
E6.5
ASPP2EpiWT/ΔE4
E6.5
ASPP2EpiΔE4/ΔE4
g
SOX17 cl. Casp-3
DAPI
E7.5
ASPP2EpiWT/ΔE4
E7.5
ASPP2EpiΔE4/ΔE4
i
SCRIB
F-actin
SCRIB F-actin
DAPI
Wild type
j'
j''
j'''
ASPP2RAKA/RAKA
f
PHH3
PHH3 F-actin
ASPP2EpiWT/ΔE4
E6.5
ASPP2EpiΔE4/ΔE4
E6.5
NS
6e−05
4e−05
2e−05
0e+00
PHH3-positive cells per µm3
ASPP2EpiWT/ΔE4
ASPP2EpiΔE4/ΔE4
3D opacity rendering
h
Cleaved Caspase-3
F-actin
ASPP2EpiWT/ΔE4
E6.5
ASPP2EpiΔE4/ΔE4
E6.5
3D opacity rendering
j
Wild type
ASPP2RAKA/RAKA
SCRIB
j'
j''
j'''

Supplementary Fig. 2 | ASPP2 is required specifically in the epiblast during proamniotic cavity formation. a, Immunofluorescence of wild type (representative image from 6 embryos) and *ASPP2*$^{\Delta E4/\Delta E4}$ (representative image from 2 embryos) E5.5 embryos using an anti-Par6 antibody. **b**, The localisation of Brachyury (T) was analysed by indirect immunofluorescence in wild type (representative image from 8 embryos) and *ASPP2*$^{\Delta E4/\Delta E4}$ (representative image from 3 embryos) embryos at E7.5. Green star: ectopic accumulation of cells in the proamniotic cavity of *ASPP2*$^{\Delta E4/\Delta E4}$ embryos. **c**, 3D opacity rendering of wild type and *ASPP2*$^{\Delta E4/\Delta E4}$ E6.5 embryos showing the localisation pattern of GATA6 and Par6 in the VE. **d**, Localisation pattern of Par6 in the EXE of wild type and *ASPP2*$^{\Delta E4/\Delta E4}$ E6.5 embryos (magnification of images presented in fig. 2a). **e**, Localisation pattern of E-cadherin in *ASPP2*$^{EpiWT/\Delta E4}$ (representative image from 4 embryos) and *ASPP2*$^{Epi\Delta E4/\Delta E4}$ E6.5 embryos (representative image from 5 embryos). Orange arrowhead: area of disrupted E-cadherin localisation. **f**, 3D opacity rendering of Phospho-Histone H3 immunostaining (PHH3) in *ASPP2*$^{EpiWT/\Delta E4}$ and *ASPP2*$^{Epi\Delta E4/\Delta E4}$ E6.5 embryos. The right panel displays the quantification of PHH3-positive cells in the epiblasts of *ASPP2*$^{EpiWT/\Delta E4}$ (n=4 embryos) and *ASPP2*$^{Epi\Delta E4/\Delta E4}$ E6.5 embryos (n=4 embryos). The total number of PHH3-positive cells per epiblast was normalised by the total volume of the epiblast. NS: non-significant (unpaired two-sided Student's T-test). **g**, SOX17 and cleaved Caspase-3 immunostaining in *ASPP2*$^{EpiWT/\Delta E4}$ and *ASPP2*$^{Epi\Delta E4/\Delta E4}$ E7.5 embryos. Orange arrowhead: cleaved Caspase-3-positive cells in the centre of the embryo where cells accumulated. **h**, 3D opacity rendering showing cleaved Caspase-3-positive cells in *ASPP2*$^{EpiWT/\Delta E4}$ and *ASPP2*$^{Epi\Delta E4/\Delta E4}$ E6.5 embryos. White arrowheads: cleaved Caspase-3-positive cells in the epiblast. **i**, SCRIB expression pattern was analysed by indirect immunofluorescence in wild type (representative images from 6 embryos) and *ASPP2*$^{RAKA/RAKA}$ (representative images from 3 embryos) embryos. The ectopic accumulations of cells in the epiblast of *ASPP2*$^{RAKA/RAKA}$ embryos was highlighted by a dashed line. **j**, Magnification of the corresponding regions in d. Blue arrowhead: enrichment of SCRIB at the apical junctions. Red arrowheads: basolateral SCRIB. The F-actin cytoskeleton and nuclei were visualised using Phalloidin and DAPI, respectively. Scale bars: 20 μm. Source data are provided as a Source Data file.

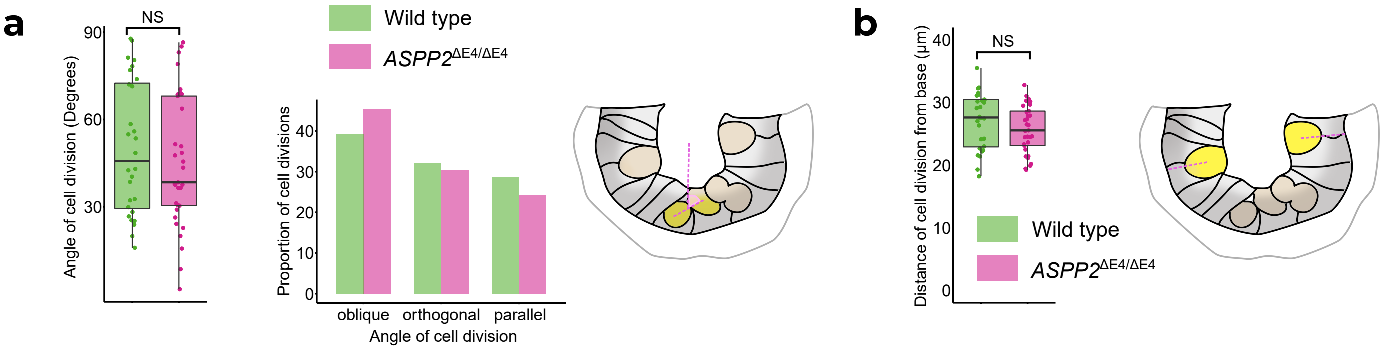

Supplementary Fig. 3 | The angle of cell division and their relative position from the base of the epiblast is unaffected in the absence of ASPP2. a, Quantification of cell division angles in the epiblasts of wild type (n=3 embryos, 28 cells) and *ASPP2*$^{\Delta E4/\Delta E4}$ embryos (n=3 embryos, 33 cells). Left panel: Comparison of all cell division angles in wild type and *ASPP2*$^{\Delta E4/\Delta E4}$ embryos. NS: non-significant (nested ANOVA). right panel: Cell division angles were defined as either parallel (0° to 30°), oblique (30° to 60°) or orthogonal (60° to 90°). NS: non-significant (two-sided Fisher's exact test of independence). **b**, Position of cell division events in wild type (n=3 embryos, 28 cells) and *ASPP2*$^{\Delta E4/\Delta E4}$ embryos (n=3 embryos, 33 cells). The relative position of cell division events was expressed as the distance between mother cell position immediately prior to a division event and the base of the epithelium. For the box plots (a,b), the top and bottom lines of each box represent the 75th and 25th percentiles, respectively. The whiskers show the minima to the maxima values and the central line indicates the median. NS: non-significant (nested ANOVA). Source data are provided as a Source Data file.

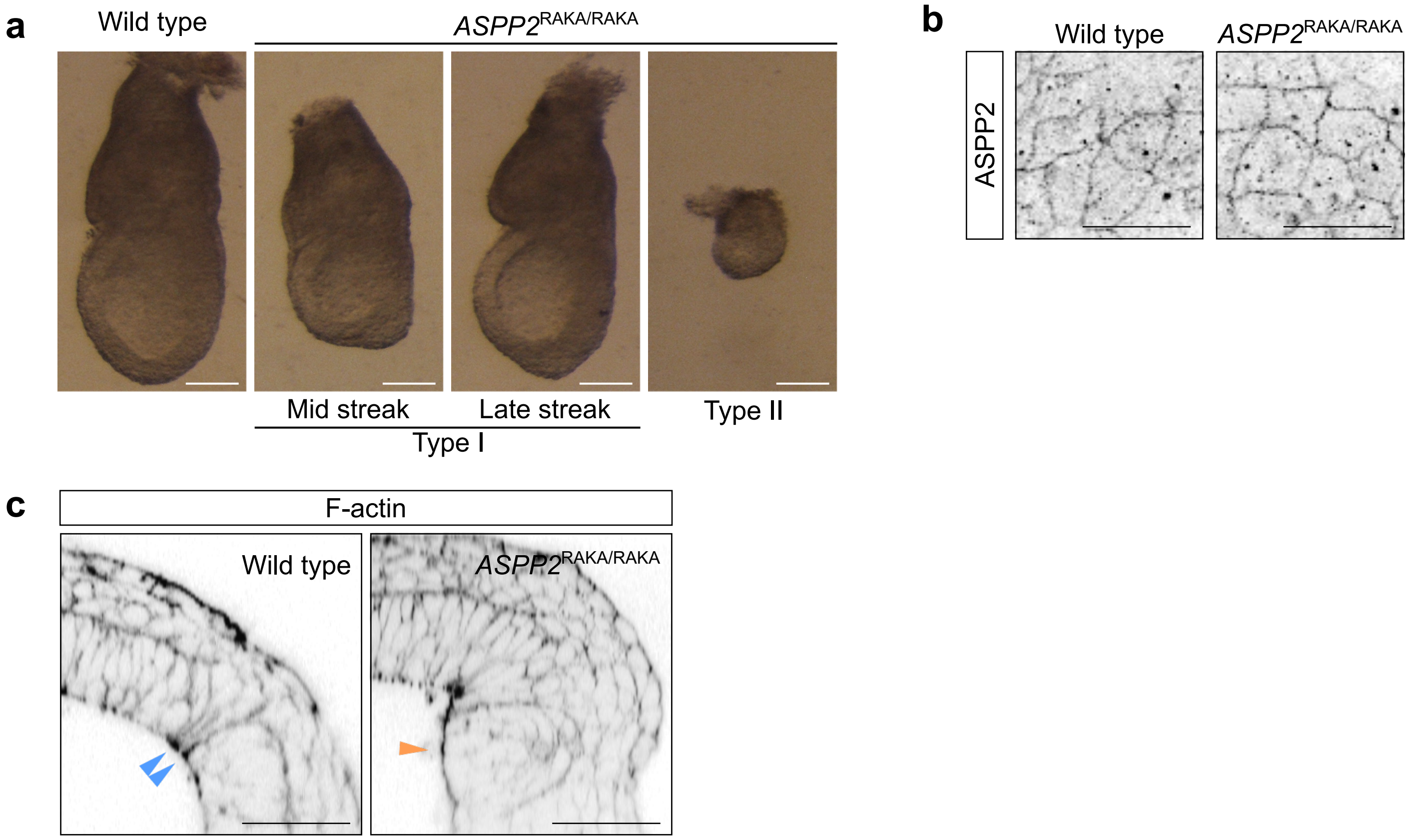

Supplementary Fig. 4 | Gross defects in *ASPP2*$^{RAKA/RAKA}$ embryos in a BALB/c background at E7.5. a, Bright field images of wild type (119) and type I (34/41, 82.9%) and type II (7/41, 17.1%) phenotypes observed in *ASPP2*$^{RAKA/RAKA}$ embryos. Type I embryos exhibited a strong accumulation of cells in their posterior. **b**, Localisation of ASPP2 and ASPP2^{RAKA} proteins at the apical junctions of the VE in wild type (representative image from 7 embryos) and *ASPP2*$^{RAKA/RAKA}$ (representative image from 5 embryos) E7.5 embryos. **c**, Cells ectopically accumulating in the primitive streak region are unable to apically constrict and do not have enriched F-actin at the apical junctions (orange arrowhead) in comparison to wild type (blue arrow heads). Representative images from 55 wild type embryos and 23 *ASPP2*$^{RAKA/RAKA}$ embryos. The F-actin cytoskeleton was visualised using Phalloidin. Scale bars: 50 μm (a and c), 20 μm (b).

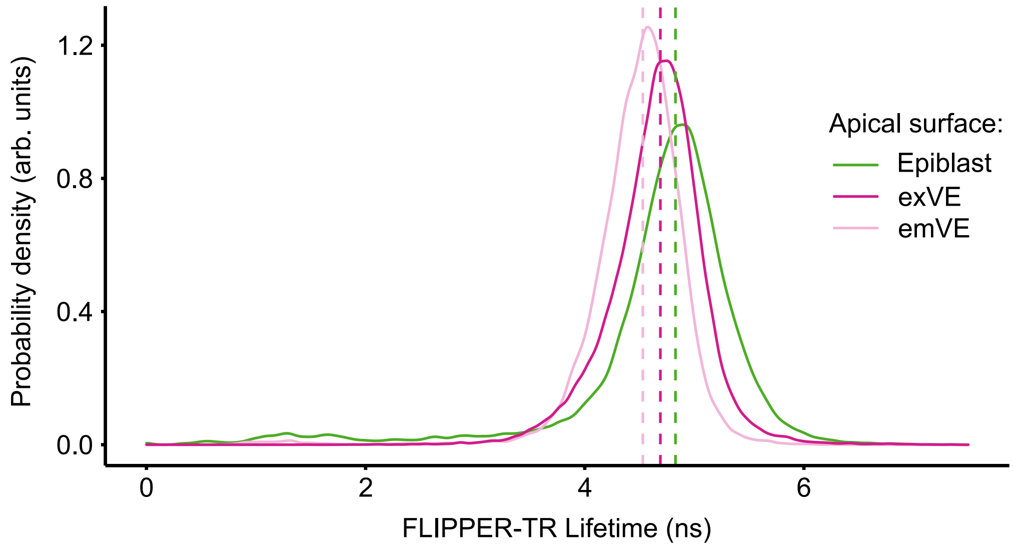

Supplementary Fig. 5 | Distribution of the FLIPPER-TR lifetimes at the apical surface of the epiblast, the emVE and the exVE in E6.5 embryos. Density plot showing the distribution of all pixel lifetime values of the FLIPPER-TR probe at the apical surface of the indicated tissues. Median pixel lifetime values are indicated with vertical dotted lines. Source data are provided as a Source Data file.

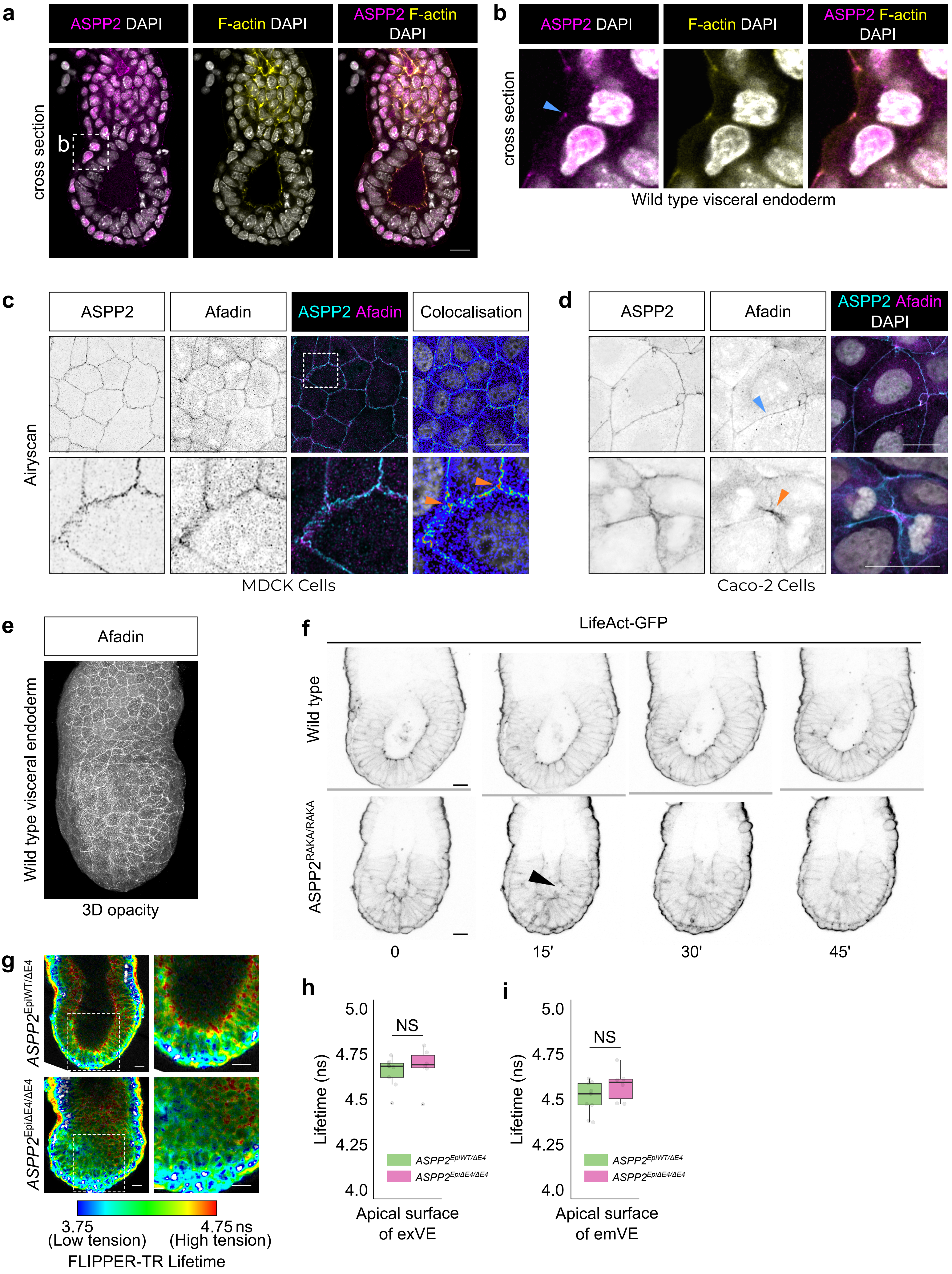

a
ASPP2 DAPI
F-actin DAPI
ASPP2 F-actin DAPI
cross section
b
b
ASPP2 DAPI
F-actin DAPI
ASPP2 F-actin DAPI
cross section
Wild type visceral endoderm
c
ASPP2
Afadin
ASPP2 Afadin
Colocalisation
Airyscan
MDCK Cells
d
ASPP2
Afadin
ASPP2 Afadin DAPI
Caco-2 Cells
e
Afadin
Wild type visceral endoderm
3D opacity
f
LifeAct-GFP
Wild type
ASPP2RAKA/RAKA
0
15'
30'
45'
g
ASPP2EpiWT/ΔE4
ASPP2EpiΔE4/ΔE4
3.75 (Low tension)
4.75 ns (High tension)
FLIPPER-TR Lifetime
h
Lifetime (ns)
5.0
4.75
4.5
4.25
4.0
NS
ASPP2EpiWT/ΔE4
ASPP2EpiΔE4/ΔE4
Apical surface of exVE
i
Lifetime (ns)
5.0
4.75
4.5
4.25
4.0
NS
ASPP2EpiWT/ΔE4
ASPP2EpiΔE4/ΔE4
Apical surface of emVE

Supplementary Fig. 6 | ASPP2 is required for the integrity of the F-actin cytoskeleton in the epiblast as cells divide. **a**, The localisation pattern of ASPP2 was analysed in wild type (representative images from 6 embryos) E5.5 embryos by immunofluorescence. **b**, Magnification of the boxed region in a, showing the localisation pattern of ASPP2 in the VE at the apical junctions (blue arrowhead). Note that ASPP2's nuclear signal is non-specific. **c**. Super-resolution Airyscan imaging of MDCK cells immunostained for ASPP2 and Afadin (representative images from three independent experiments). Orange arrowheads highlight the colocalisation of Afadin and ASPP2 at tricellular junctions. **d**, The localisation pattern of ASPP2 and Afadin was analysed in Caco-2 cells (representative images from 5 independent experiments). Afadin and ASPP2, in addition to being enriched at tricellular junctions, could also be found colocalising at bicellular junctions (blue arrowhead) and were in close proximity at the cleavage furrow (orange arrowhead). **e**, 3D opacity rendering showing the localisation of Afadin at the apical junctions of the VE in an E6.5 wild type embryo. **f**, Time-lapse imaging of wild type and *ASPP2*$^{RAKA/RAKA}$ LifeAct-GFP positive embryos. Note how apical F-actin is disrupted in *ASPP2*$^{RAKA/RAKA}$ LifeAct-GFP positive embryos following a cell division event (black arrowhead). Nuclei and the F-actin cytoskeleton were visualised with DAPI and Phalloidin, respectively. Scale bars: 20 μm. **g**, Representative FLIM images of *ASPP2*$^{EpiWT/\Delta E4}$ (n=9 embryos) and *ASPP2*$^{Epi\Delta E4/\Delta E4}$ (n=7 embryos) embryos. Magnifications of the regions delimited with dotted lines are represented. Scale bars: 20 μm. **h**,**i**, Quantification of mean lifetime values in *ASPP2*$^{EpiWT/\Delta E4}$ (n=9 embryos) and *ASPP2*$^{Epi\Delta E4/\Delta E4}$ (n=7 embryos) embryos at the apical surface of the exVE (g) and emVE (h). For the box plots (h,i), the top and bottom lines of each box represent the 75th and 25th percentiles, respectively. The whiskers show the minima to the maxima values and the central line indicates the median. Outliers are represented with black dots. NS: not significant (unpaired two-sided Student's T-test). Source data are provided as a Source Data file.

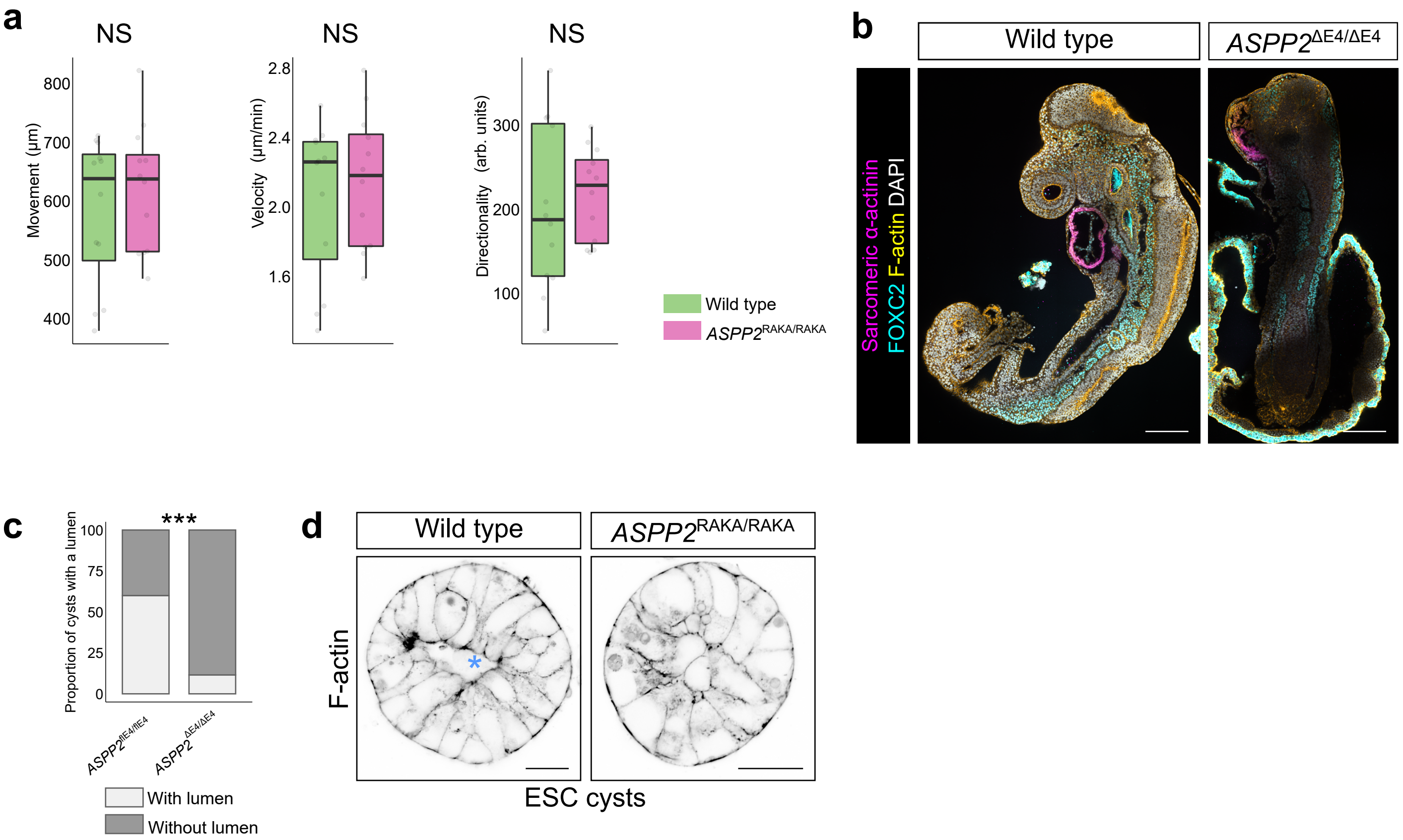

Supplementary Fig. 7 | General required for ASPP2 across pseudostratified epithelia. a, Mesoderm cell migration is unaffected in the absence of ASPP2. Cell movement, velocity, and directionality from wild type (n=4 explants from individual embryos, 3 cells per explant) and *ASPP2*$^{RAKA/RAKA}$ (n=4 explants from individual embryos, 3 cells per explant) mesoderm explants were quantified. For the box plots, the top and bottom lines of each box represent the 75th and 25th percentiles, respectively. The whiskers show the minima to the maxima values and the central line indicates the median. NS: non-significant (nested ANOVA). **b**, E9.5 wild type and *ASPP2*$^{\Delta E4/\Delta E4}$ embryos labelled by immunofluorescence with antibodies against FOXC2 (somitic mesoderm) and sarcomeric α-actinin (sarcomeres in cardiomyocytes). Nuclei and the F-actin cytoskeleton were visualised with DAPI and Phalloidin, respectively. **c**, Quantification of the proportion of *ASPP2*$^{flE4/flE4}$ (n=25 cysts) and *ASPP2*$^{\Delta E4/\Delta E4}$ ESC-derived cysts (n=26 cysts) with lumens after three days in culture in Matrigel. *** p<0.001 (two-sided Fisher's exact test of independence, p=3.89e-4). **d**, Representative images from three independent experiments of wild type and *ASPP2*$^{RAKA/RAKA}$ ESC-derived cysts after three days in culture in Matrigel. The blue asterisk indicates the lumen. The F-actin cytoskeleton was visualised with Phalloidin, respectively. Scale bars: 200 µm (b), 20 µm (d). Source data are provided as a Source Data file.
