# Supplementary material for: ASPP2 maintains the integrity of mechanically stressed pseudostratified epithelia during morphogenesis
Source: Nat Commun. 2022 Feb 17;13:941. doi: 10.1038/s41467-022-28590-4 (PMC8854694; doi:10.1038/s41467-022-28590-4)
Supplement: Supplementary file 3 — Description of Additional Supplementary Files [file 41467_2022_28590_MOESM3_ESM.pdf]

Description of Additional Supplementary Files

File Name: Supplementary Movie 1
Description: **Time lapse imaging of wild type and ASPP2ΔE4/ΔE4 embryos with mT/mGlabelled cell membranes**

File Name: Supplementary Movie 2
Description: **Airyscan imaging and 3D rendering of F-actin in the primitive streak region of representative wild type and ASPP2RAKA/RAKA embryos**

File Name: Supplementary Movie 3
Description: **3D opacity rendering showing that the primitive streak expands comparatively in E7.5 wild type and ASPP2ΔE4/ΔE4 embryos.** Mesoderm cells were labelled by immunofluorescence using an antibody against Brachyury (T). Nuclei and the F-actin cytoskeleton were visualised with DAPI and Phalloidin, respectively.

File Name: Supplementary Movie 4
Description: **Time lapse imaging of wild type and ASPP2RAKA/RAKA mesoderm explants positive for the LifeAct-GFP transgene**
