# Supplementary material for: ASPP2 maintains the integrity of mechanically stressed pseudostratified epithelia during morphogenesis
Source: Nat Commun. 2022 Feb 17;13:941. doi: 10.1038/s41467-022-28590-4 (PMC8854694; doi:10.1038/s41467-022-28590-4)
Supplement: Supplementary file 8 — Reporting Summary [file 41467_2022_28590_MOESM8_ESM.pdf]

nature portfolio

Corresponding author(s): Christophe Royer and Shankar Srinivas

Last updated by author(s): Jan 26, 2022

Reporting Summary

Nature Portfolio wishes to improve the reproducibility of the work that we publish. This form provides structure for consistency and transparency in reporting. For further information on Nature Portfolio policies, see our Editorial Policies and the Editorial Policy Checklist.

Statistics

For all statistical analyses, confirm that the following items are present in the figure legend, table legend, main text, or Methods section.

n/a	Confirmed	
☐	☒	The exact sample size (*n*) for each experimental group/condition, given as a discrete number and unit of measurement
☐	☒	A statement on whether measurements were taken from distinct samples or whether the same sample was measured repeatedly
☐	☒	The statistical test(s) used AND whether they are one- or two-sided<br>*Only common tests should be described solely by name; describe more complex techniques in the Methods section.*
☒	☐	A description of all covariates tested
☒	☐	A description of any assumptions or corrections, such as tests of normality and adjustment for multiple comparisons
☐	☒	A full description of the statistical parameters including central tendency (e.g. means) or other basic estimates (e.g. regression coefficient) AND variation (e.g. standard deviation) or associated estimates of uncertainty (e.g. confidence intervals)
☐	☒	For null hypothesis testing, the test statistic (e.g. *F*, *t*, *r*) with confidence intervals, effect sizes, degrees of freedom and *P* value noted<br>*Give P values as exact values whenever suitable.*
☒	☐	For Bayesian analysis, information on the choice of priors and Markov chain Monte Carlo settings
☒	☐	For hierarchical and complex designs, identification of the appropriate level for tests and full reporting of outcomes
☒	☐	Estimates of effect sizes (e.g. Cohen's *d*, Pearson's *r*), indicating how they were calculated

Our web collection on statistics for biologists contains articles on many of the points above.

Software and code

Policy information about availability of computer code

Data collection	No custom computer code was used for data collections.
Data analysis	The following softwares were used for data collection/analysis: Volocity (version 6.3.1, PerkinElmer), Zen (Zeiss), Fiji (ImageJ 1.53j), LAS-X (Leica Microsystems), R version 3.6.0 and RStudio Version 1.2.1335, Imaris v.6.3. The following plugins were used in Fiji: TrackMate (v5.2.0), Manual Tracking.<br>For quantitative analysis the Phasor-FLIM images were exported to .tiff (using 0.01 lifetime values per grey level to ensure accuracy). The .tiff-files contained the intensity images as well as the lifetime images and were further processed in Fiji using a custom written macro available at https://github.com/Faldalf/Royer_et_al_FLIM_ROIs.git.

For manuscripts utilizing custom algorithms or software that are central to the research but not yet described in published literature, software must be made available to editors and reviewers. We strongly encourage code deposition in a community repository (e.g. GitHub). See the Nature Portfolio guidelines for submitting code & software for further information.

Data

Policy information about availability of data

All manuscripts must include a data availability statement. This statement should provide the following information, where applicable:

- Accession codes, unique identifiers, or web links for publicly available datasets
- A description of any restrictions on data availability
- For clinical datasets or third party data, please ensure that the statement adheres to our policy

The complete data supporting the results presented in this study are available upon a reasonable request from the corresponding authors. Source data are provided with this paper.

Field-specific reporting

Please select the one below that is the best fit for your research. If you are not sure, read the appropriate sections before making your selection.

[x] Life sciences [ ] Behavioural & social sciences [ ] Ecological, evolutionary & environmental sciences

For a reference copy of the document with all sections, see nature.com/documents/nr-reporting-summary-flat.pdf

Life sciences study design

All studies must disclose on these points even when the disclosure is negative.

Sample size	No sample size calculation was performed. For a given experiment and developmental stage, the number of embryos used for each genotype (control and experimental) was generally at least three (or more), which is commonly accepted best practice in mouse embryo studies and in the field of developmental biology.
Data exclusions	Embryos visibly damaged during dissection were excluded from our study.
Replication	For all analyses involving embryos, findings were replicated in at least three individual embryos. For other types of experiments, at least three independent experiments were conducted. Similar results were required in all biological replicates to support or reject a given hypothesis. All attempts at replication confirmed the reported findings.
Randomization	Randomization of the samples is not applicable to our study, since no treatment conditions were compared. All comparisons were performed between different genotypes, which do not require randomization.
Blinding	The investigators were not blinded to group allocation. Blinding was not possible because the phenotype was clearly visible in most cases.

Reporting for specific materials, systems and methods

We require information from authors about some types of materials, experimental systems and methods used in many studies. Here, indicate whether each material, system or method listed is relevant to your study. If you are not sure if a list item applies to your research, read the appropriate section before selecting a response.

Materials & experimental systems

n/a	Involved in the study	
[ ]	[x]	Antibodies
[ ]	[x]	Eukaryotic cell lines
[x]	[ ]	Palaeontology and archaeology
[ ]	[x]	Animals and other organisms
[ ]	[x]	Human research participants
[x]	[ ]	Clinical data
[x]	[ ]	Dual use research of concern

Methods

n/a	Involved in the study	
[x]	[ ]	ChIP-seq
[x]	[ ]	Flow cytometry
[x]	[ ]	MRI-based neuroimaging

Antibodies

Antibodies used	rabbit anti-ASPP2 (Sigma, HPA021603), 1:100-1:200 (IHC); mouse anti-ASPP2 (Santa Cruz Biotechnologies, sc135818), 1:100 (ICC), 1:1000 (IB); mouse anti-YAP (Santa Cruz Biotechnology, sc-101199), 1:100 (IHC); rabbit anti-pYAP S127 (Cell Signaling, 4911), 1:100 (IHC); rabbit anti-Par3 (Millipore, 07-330), 1:100 (IHC); rabbit anti-Pard6b (Santa Cruz Biotechnology, sc-67393), 1:100 (IHC); rabbit anti-SCRIB (Santa Cruz Biotechnology, sc28737), 1:100 (IHC); rat anti-E-cadherin (Sigma, U3254), 1:100 (IHC); goat anti-SOX17 (R&D Systems, AF1924), 1:100 (IHC); rabbit anti-Phospho-Histone H3 (Cell Signaling, 9713), 1:200 (IHC); rabbit anti-Cleaved Caspase-3 (Cell Signaling, 9661), 1:100 (IHC); goat anti-Brachyury (Santa Cruz Biotechnology, sc17745), 1:100 (IHC); rabbit anti-Sarcomeric α-actinin (Abcam, ab68167), 1:100 (IHC); mouse anti-FOXC2 (Santa Cruz Biotechnology, sc515234), 1:100 (IHC); rabbit anti-SOX-2 (Millipore, AB5603), 2 µl per mg of cell lysate (co-IP), 1:100 (IHC); goat anti-NKX2.5 (Santa Cruz Biotechnology, sc8697), 1:100 (IHC); rabbit anti-Afadin (Sigma, A0224), 2 µl per mg of cell lysate (co-IP), 1:100 (IHC, ICC), 1:1000 (IB); rabbit anti-Laminin (Sigma, L9393), 1:200 (IHC); goat anti-AMOT (Santa Cruz Biotechnologies, sc82491), 1:200 (IHC); goat anti-GATA-6 (R&D Systems, AF1700), 1:100 (IHC); rabbit anti-Myosin IIa (Cell Signaling, #3403), 1:100; rabbit anti-phospho-Myosin light chain 2 (Cell Signaling, #3674), 1:100. The following were used at 1:100 for IHC and 1:400 for ICC: Alexa fluor 555 donkey-anti-mouse (Invitrogen, A-31570), Alexa fluor 647 goat-anti-rat (Invitrogen, A-21247), Alexa fluor 488 donkey-anti-rabbit (Invitrogen, A21206).
Validation	The specificity of the rabbit anti-ASPP2 antibody (Sigma, HPA021603) was tested in embryos by comparing the signal observed in control epiblasts versus epiblasts in which ASPP2 was deleted (Fig. 3c). Note that the ASPP2 antibody results in non-specific nuclear signal (also seen in Fig. 1c when depleting ASPP2 by siRNA).<br><br>All other antibodies have previously been validated and used in multiple publications that are referenced on the manufacturers 'websites:

mouse anti-ASPP2 (Santa Cruz Biotechnologies, sc135818): validated for ICC and IB in Royer et al., Plos One, 2014
mouse anti-YAP(Santa Cruz Biotechnology, sc-101199): validated in Hirate et al., Current Biology, 2013 (mouse pre-implantation embryos, wholemount IHC)
rabbit anti-pYAP S127 (Cell Signaling, 4911): validated in Hirate et al., Current Biology, 2013 (mouse pre-implantation embryos, wholemount IHC)
rabbit anti-Par3 (Millipore, 07-330): validated in Sottocornola et al., Developmental Cell, 2010 (mouse tissues, IHC)
rabbit anti-Pard6b (Santa Cruz Biotechnology, sc-67393): validated in Hirate et al., Current Biology, 2013 (mouse pre-implantation embryos, wholemount IHC)
rabbit anti-SCRIB (Santa Cruz Biotechnology, sc28737): validated in Hirate et al., Current Biology, 2013 (mouse pre-implantation embryos, wholemount IHC)
rat anti-E-cadherin (Sigma, U3254): selected against the mouse cell adhesion molecule uvomorulin/E-Cadherin and validated using MDCK cells. Works in a wide range of applications (https://www.sigmaaldrich.com/GB/en/product/sigma/u3254)
goat anti-SOX17 (R&D Systems, AF1924): validated in a wide range of applications and species, including in mouse tissue IHC (https://www.rndsystems.com/products/human-sox17-antibody_af1924#product-citations)
rabbit anti-Phospho-Histone H3 (Cell Signaling, 9713): validated in a wide range of applications and species, including in mouse tissue IHC (https://www.cellsignal.co.uk/products/primary-antibodies/phospho-histone-h3-ser28-antibody/9713)
rabbit anti-Cleaved Caspase-3 (Cell Signaling, 9661): validated in a wide range of applications and species, including in mouse tissue IHC (https://www.cellsignal.co.uk/products/primary-antibodies/cleaved-caspase-3-asp175-antibody/9661)
goat anti-Brachyury (Santa Cruz Biotechnology, sc17745): validated in wide range of applications and species (https://www.scbt.com/p/brachyury-antibody-c-19), including in mouse embryos in Van Eynde et al., Molecular and Cellular Biology, 2004
rabbit anti-Sarcomeric aplha-actinin (Abcam, ab68167): validated in a wide range of applications and species, including in mouse tissue IHC (https://www.abcam.com/sarcomeric-alpha-actinin-antibody-ep2529y-ab68167.html)
mouse anti-FOXC2 (Santa Cruz Biotechnology, sc515234): validated in a wide range of applications and species, including in mouse tissue IHC (https://www.scbt.com/p/foxc2-antibody-g-7)
rabbit anti-SOX-2 (Millipore, AB5603): validated in mouse tissue IHC (https://www.merckmillipore.com/GB/en/product/Anti-Sox2-Antibody,MM_NF-AB5603)
goat anti-NKX2.5 (Santa Cruz Biotechnology, sc8697): validated in a wide range of applications and species, including in mouse tissue IHC (https://www.scbt.com/p/nkx-2-5-antibody-n-19)
rabbit anti-Afadin (Sigma, A0224): validated in Yang et al., Development, 2013 (mouse tissue, IHC)
rabbit anti-Laminin (Sigma, L9393): validated in mouse tissue IHC (https://www.sigmaaldrich.com/GB/en/product/sigma/l9393)
goat anti-AMOT (Santa Cruz Biotechnologies, sc82491): validated in Hirate et al., Current Biology, 2013 (mouse pre-implantation embryos, wholemount IHC)
goat anti-GATA-6 (R&D Systems, AF1700): validated in mouse tissue IHC (https://www.rndsystems.com/products/human-gata-6-antibody_af1700#product-citations)
rabbit anti-Myosin IIa (Cell Signaling, #3403): validated in mouse tissue IHC (https://www.cellsignal.co.uk/products/primary-antibodies/myosin-iia-antibody/3403)
rabbit anti-phospho-Myosin light chain 2 (Cell Signaling, #3674): validated in mouse tissue IHC (https://www.cellsignal.co.uk/products/primary-antibodies/phospho-myosin-light-chain-2-thr18-ser19-antibody/3674)

Eukaryotic cell lines

Policy information about cell lines

Cell line source(s)	Caco-2 cells were obtained from Professor Xin Lu.<br>MDCKII cells were a kind gift from Professor Manuela Zaccolo.<br>We could not trace back the commercial source for these cell lines but Caco-2 cells are available from ATCC (https://www.atcc.org/products/htb-37) and MDCKII cells from ECACC (https://www.phe-culturecollections.org.uk/products/celllines/generalcell/detail.jsp?refId=00062107&collection=ecacc_gc)<br>Mouse ESC lines were derived from blastocysts as described in the Materials and Methods section of the mansucript.
Authentication	None of the cell lines used were authenticated.
Mycoplasma contamination	All cell lines were free of mycoplasma contamination as tested using DAPI.
Commonly misidentified lines (See ICLAC register)	No commonly misidentified cell lines were used in the study

Animals and other organisms

Policy information about studies involving animals; ARRIVE guidelines recommended for reporting animal research

Laboratory animals	Laboratory animals used in this study are of the species Mus musculus (CD1, C57BL/6J, C57BL/6n and BALB/cOlaHsd strains). For natural matings, sexually mature females were used (usually 6 week to 3 month-old). For superovulations, 3 to 4 week old CD-1 females were used.
Wild animals	This study did not use any wild animals.
Field-collected samples	This study did not use any field-collected samples.
Ethics oversight	All animal experiments complied with the UK Animals (Scientific Procedures) Act 1986, were approved by the local Biological Services Ethical Review Process and were performed under UK Home Office project licenses PPL 30/3420 and PCB8EF1B4. The LERP (local

ethical review panel) at the Department of Physiology, Anatomy and Genetics approved the study.

Note that full information on the approval of the study protocol must also be provided in the manuscript.

Human research participants

Policy information about studies involving human research participants

Population characteristics	Early human pre-implantation embryos obtained from IVF were used in this study. These embryos were at the blastocyst stage. No information on their gender was collected.<br>We don't have information on patient age etc as this type of info is blinded from the researchers.
Recruitment	All new patients intending to come to the unit for fertility treatment were given an information pack when they attended the evening meeting before starting treatment. An Information sheet about research projects using surplus eggs and embryos was included in the pack. Patients would not typically visit the clinic until several weeks after receiving this, giving time for them to consider whether or not they want to participate. All patients commencing their fertility treatment then arranged a routine new patient consultation appointment. At this visit doctors/nurses woulkd check that the patient meets the inclusion criteria to participate in the study. This includes checking that the patient has, in a questionnaire supplied to ALL patients by the HFEA (Form WT), agreed in principle to being approached about research projects involving their gametes (eggs). If so, they would ask the patient if they wanted to participate in the study. A research nurse would always be available for further discussion of the projects if necessary.
Ethics oversight	Human embryos were donated from patients attending the Oxford Fertility with approval from the Human Fertilization and Embryology Authority (Centre 0035, project RO198) and the Oxfordshire Research Ethics Committee (NRES Committee South Central – Berkshire B; Reference number 14/SC/0011). Informed consent was attained from all patients. The study design and conduct complied with all relevant regulations regarding the use of human study participants and was conducted in accordance with the criteria set by the Declaration of Helsinki.

Note that full information on the approval of the study protocol must also be provided in the manuscript.
